# Estimating and predicting snakebite risk in the Terai region of Nepal through a high-resolution geospatial and One Health approach

**DOI:** 10.1038/s41598-021-03301-z

**Published:** 2021-12-13

**Authors:** Carlos Ochoa, Marta Pittavino, Sara Babo Martins, Gabriel Alcoba, Isabelle Bolon, Rafael Ruiz de Castañeda, Stéphane Joost, Sanjib Kumar Sharma, François Chappuis, Nicolas Ray

**Affiliations:** 1 Institute of Global Health (IGH), Department of Community Health and Medicine, Faculty of Medicine, University of Geneva, Chemin des Mines 9, 1202 Geneva, Switzerland; 2 Institute for Environmental Sciences (ISE), University of Geneva, Geneva, Switzerland; 3 Research Center for Statistics (RCS), Geneva School of Economics and Management (GSEM), University of Geneva, Geneva, Switzerland; 4 Médecins Sans Frontières (MSF), Geneva, Switzerland; 5 Division of Tropical and Humanitarian Medicine, Geneva University Hospitals (HUG), Geneva, Switzerland; 6 Laboratory of Geographic Information Systems (LASIG), School of Architecture, Civil and Environmental Engineering (ENAC), École Polytechnique Fédérale de Lausanne (EPFL), Lausanne, Switzerland; 7 B.P. Koirala Institute of Health Sciences (BPKIHS), Dharan, Nepal; 8 Department of Community Health and Medicine, Faculty of Medicine, University of Geneva, Geneva, Switzerland

**Keywords:** Ecological epidemiology, Risk factors, Epidemiology

## Abstract

Most efforts to understand snakebite burden in Nepal have been localized to relatively small areas and focused on humans through epidemiological studies. We present the outcomes of a geospatial analysis of the factors influencing snakebite risk in humans and animals, based on both a national-scale multi-cluster random survey and, environmental, climatic, and socio-economic gridded data for the Terai region of Nepal. The resulting Integrated Nested Laplace Approximation models highlight the importance of poverty as a fundamental risk-increasing factor, augmenting the snakebite odds in humans by 63.9 times. For animals, the minimum temperature of the coldest month was the most influential covariate, increasing the snakebite odds 23.4 times. Several risk hotspots were identified along the Terai, helping to visualize at multiple administrative levels the estimated population numbers exposed to different probability risk thresholds in 1 year. These analyses and findings could be replicable in other countries and for other diseases.

## Introduction

Currently, it is estimated that 50 people worldwide are bitten by a snake every five minutes, and one of them will die^1^. From the approximate 5.4 million people bitten by snakes globally every year, up to 2.7 million are envenomed, leading to more than 400,000 disability cases and between 81,000 and 138,000 deaths^2–4^. Affected populations often lack appropriate access to healthcare^5^. In addition to the devastation caused to individuals, families, and communities, snakebite envenoming also causes important losses of livestock^6^, which have substantial livelihood impacts for the affected households^7^.

In rural, agricultural populations, poverty has been extensively documented as a crucial factor linked to high snakebite incidence. Indeed, Nepal is a primarily agricultural country, with more than 65% of the population working in agriculture^8^. More than 50% of its population lives in the Terai region, the south plains^9^ where most of Nepal’s agricultural activity is concentrated^10^. This area is characterized by a high snakebite incidence both in humans and animals, recently estimated at 261 cases per 100,000 people a year, and up to 202 cases per 100,000 animals a year depending on species^11^.

In Nepal, as in many endemic countries, both the public and the medical personnel lack awareness of and education about snakebite, which hinders victims in seeking appropriate medical treatment^10^. Regarding domestic animals, it is even rarer for owners to seek and access veterinary services that can treat snakebite, relying more often on traditional medicine^12^.

Geospatial approaches have recently allowed direct and indirect estimation of the present^13–16^ and future^17^ risk of snakebite and other spatially distributed health and social problems^18,19^. However, countrywide studies using spatial epidemiology, as done in Bangladesh^20^ and Sri Lanka^21^, still remain rare despite being invaluable to understand the burden of snakebite at national scale. Similar methods from a One Health perspective, incorporating the risks and consequences of snakebite for domestic animals, are almost non-existent^6^. Until recently, previous studies in Nepal analysed the incidence of snakebite at district and sub-district level, either as community survey^22^ or based on medical records^23,24^, but none have addressed the incidence or risk of snakebite at a national or subnational scale. Recently, human and animal snakebite incidences were analysed nationally for the Terai^11,12^, and here we analyse geospatially the risk of snakebite and the factors influencing it in humans and animals.

The World Health Organisation and the international scientific community have recognized the incompleteness of the data associated with snakebite^4,25^, emphasizing the need to understand its intrinsic risk and to inform relevant stakeholders about it. It has been also emphasized that further research on ecological^26^ and environmental risk factors is necessary to develop better snakebite risk-reduction strategies, especially in places where other measures cannot be readily implemented^4^. Generating this knowledge at high spatial resolution is a keystone for further actions in education, prevention, accessibility to treatment, allocation of resources, community empowerment, and health systems reinforcement in Nepal and other endemic countries.

In this study, we applied a hierarchical Bayesian, Integrated Nested Laplace Approximation (INLA) methodology to spatially analyse snakebite risk in a model with two variations. In the first, we estimated the factors influencing snakebite risk for humans and domestic animals using both highly granular data from a recent Terai-wide multi-cluster random survey^27^ and geospatial gridded layers of environmental, geographic and socio-economic factors. In the second variation, and considering that the covariates from the aforementioned survey could not be imputed to any place in the Terai, we used exclusively geospatial gridded layers not only to estimate, but also to predict the snakebite risk for humans at high spatial resolution.

## Results

In total, 12,998 observations (households), were available and contributed to valid data, including 154 (1.18%) human snakebite cases, and 91 (0.7%) domestic animal cases (all species). From the 249 clusters surveyed, 15 could not be included in the analysis due to unmatched geographic coordinates. For the full epidemiological analyses in humans and animals, see Alcoba et al.^11^ and Bolon et al.^12^.

### Modelling of snakebite risk in humans

The covariates selected for the final model of snakebite risk in humans were: *food storage*, s*traw storage*, and *sleeping on the floor*, as well as poverty, quantified using the *Poverty Probability Index (PPI)*^28^, the average annual *Normalized Difference Vegetation Index (NDVI)*, and *distance to water*. These covariates, together with the spatial random effects, were integrated in a hierarchical Bayesian logistic model (see “Methods”) to estimate the marginal posterior distributions of the single parameters and hyperparameters (see Table 1), and joint posterior distributions for each observed point.

**Table 1:** Estimated parameters of the fitted hierarchical Bayesian models for the human and the animal risk of snakebite in the Terai.

	Mean	SD	Mode	Odds ${(e}^{\mathrm{Mean}})$	Odds 90% CI	
					LL	UL
**Human model**						
(Intercept)	− 2.21	0.71	− 2.20	0.11	0.03	0.35
Food storage	1.02	0.39	0.97	2.78	1.50	5.43
Straw storage	0.58	0.21	0.57	1.78	1.26	2.53
Sleeping on the floor	− 0.77	0.43	− 0.71	0.46	0.22	0.91
PPI/100	4.16	0.61	4.18	63.88	22.98	172.13
NDVI	− 1.43	0.79	− 1.41	0.24	0.06	0.87
Distance to Water (km)	0.32	0.18	0.32	1.38	1.02	1.85
Range for SPDE (ρ)	31.57	11.69	26.09			
SD for SPDE (σ)	0.97	0.16	0.94			
**Animal model**						
(Intercept)	− 5.38	0.91	− 5.36	0	0	0.02
Animal shed	1.84	0.58	1.71	6.28	2.60	17.36
Straw storage	0.50	0.28	0.48	1.64	1.04	2.65
HMTS	− 2.01	1.53	− 1.97	0.13	0.01	1.61
BIO6/10	3.15	1.62	3.09	23.41	1.68	348.09
Pig density/1000	0.82	0.44	0.87	2.27	1.07	4.52
Sheep density/1000	2.09	0.78	2.11	8.06	2.21	28.35
Range for SPDE (ρ)	432.44	366.35	208.49			
SD for SPDE (σ)	0.77	0.35	0.59			

Reported statistics are the posterior marginal mean, standard deviation and mode (logit scale), as well as the corresponding mean, 90% lower- and upper-limit credible interval (odds scale). The bottom rows in each model report the spatial random effects hyperparameters. Other abbreviations: Human modification of terrestrial systems (HMTS), minimum temperature of the coldest month (BIO6), Stochastic Partial Differential Equations (SPDE).

The model selection process led to a set of covariates, whose estimated coefficients fell, in all cases, into 90% credible intervals (CI) that did not include 1 (odds scale). Hence, they are considered significant and strongly associated with the risk of snakebite in humans. The marginal posterior distributions of all coefficients, their mean, and 90% CI are plotted in the supplementary Fig. S2.

The covariate with the strongest effect on the snakebite risk was the *PPI*, which by a change of one unit (its whole range) increased the odds of snakebite in a household 63.88 times (90% CI 22.98–172.13). *Food storage* was the second most important variable that increased the risk of snakebite by 2.78 times (90% CI 1.50–5.43), *straw storage* by 1.78 times (90% CI 1.26–2.53), and each additional unit (km) in the *distance to water* by 1.38 times (90% CI 1.02–1.85). In contrast, *NDVI* had the strongest ‘protective’ effect, with an odds ratio of 0.24 (90% CI 0.06–0.87), equivalent to a reduction of 4.36 times the odds of snakebite. Likewise, with an odds ratio of 0.46 (90% CI 0.22–0.91) *sleeping on the floor* had a significant effect in the snakebite odds with a reduction equivalent to 2.17 times. The estimated range ρ was 31.57 km, which represents the distance at which the spatial correlation between any two points becomes negligible. None of the relevant pairs of covariate interactions tested were statistically significant or helped explaining the changes in the response. The final model, with a Watanabe-Akaike Information Criterion^29^ (WAIC) of 1503.95, was also run without the spatial random component with a clear worsening of the model fit (WAIC: 1589.45), which verified the importance of this component.

### Modelling of snakebite risk in domestic animals

The selected covariates for the final snakebite risk model in animals were: *animal shed*, *straw storage*, *human modification of terrestrial systems* (0–1 metric index reflecting a cumulative measure of human modification of landscapes^30^), the *minimum temperature of the coldest month* (WorldClim BIO6), as well as *pig density* and *sheep density*^31^. The coefficients and CI for parameters and hyperparameters are shown in Table 1. Here also, the interactions evaluated were neither statistically significant nor helped explain the changes in the response variable, and therefore were not used.

The selected covariates for the final animal model were also all significant, excepting *Human modification of terrestrial systems*, which nevertheless with an odds ratio of 0.13 (90% CI 0.01–1.61) represented a reduction of the snakebite odds equivalent to 7.69 times. In contrast, all other covariates increased the odds of snakebite. The strongest effect was from *BIO6*, where a 10 °C temperature change represented an increase of 23.41 times the odds of snakebite (90% CI 1.68–348.09). *Sheep density* followed with an 8.06 times odd’s increase for each 1000 sheep (90% CI 2.21–28.35), and *animal shed* with 6.28 times odd’s increase (90% CI 2.6–17.36). *Pig density* and *straw storage* had a less strong effect, yet they increased the odds of snakebite by 2.27 times (90% CI 1.07–4.52) and 1.64 times (90% CI 1.04–2.65), respectively. For the animal model, the estimated range ρ was 432.4 km, showing a much larger spatial correlation between points. The marginal posterior distributions of the estimated parameters and hyperparameters, their mean, and 90% CI can be found in the supplementary Fig. S3. This model (WAIC: 961.54) was also tested without the spatial random component, leading to a reduction in the model fit (WAIC: 971.19).

### Mapping of snakebite risk and population at risk

**Figure 1:**
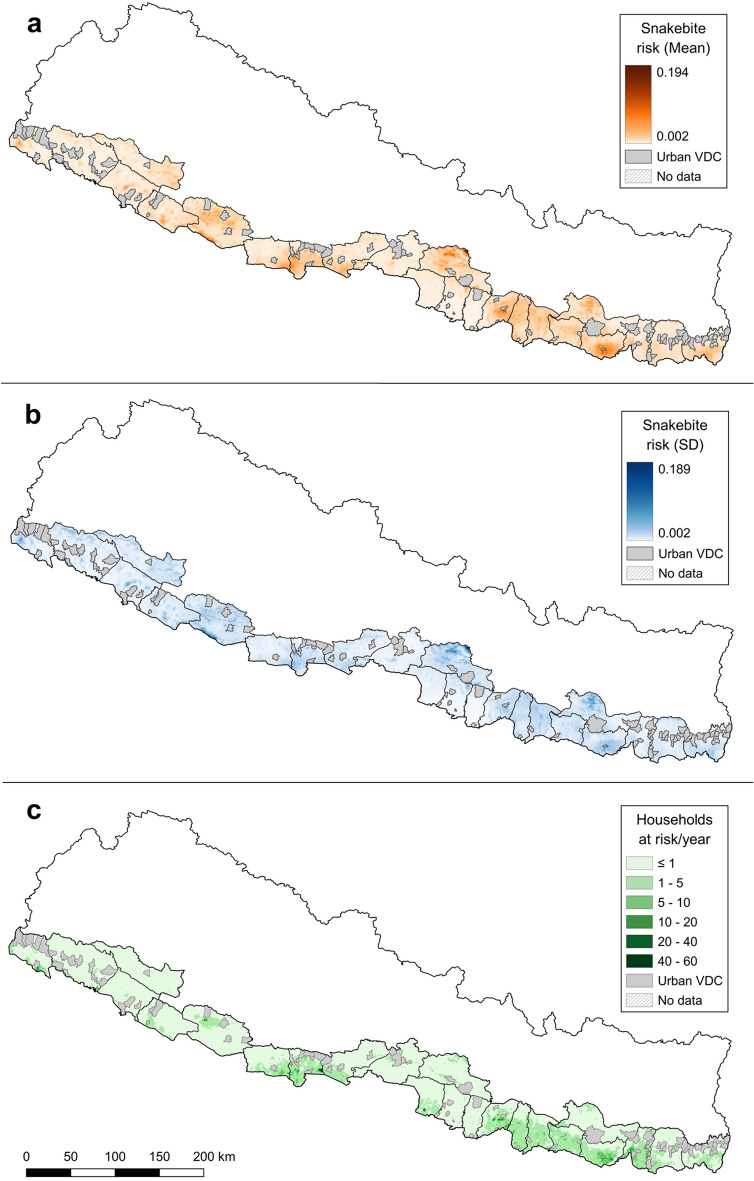
(**a**) Mean posterior distribution, (**b**) uncertainty (SD) of the snakebite risk for the Terai at 1 km^2^ resolution, and (**c**) estimated number of households at risk of snakebite per 1 km^2^/year, based on the WorldPop UN-adjusted population estimates for Nepal in 2018. (Source: vector map and administrative divisions from https://gadm.org/download_country_v3.html, projected in the local WGS 84/UTM zone 45 N coordinate reference system in QGIS 3.18.3 (https://qgis.org/en/site/)).

High-resolution gridded maps (1 km^2^) of the mean snakebite-risk predicted values, their uncertainty (SD) and the estimated average number of households at risk of snakebite per year are shown in Fig. 1. The mean risk map in Fig. 1A shows several areas with increased snakebite risk along the Terai, which roughly correspond to the areas with the highest uncertainty in Fig. 1B. The map of households at risk of snakebite (Fig. 1C) shows two main areas with high numbers of affected population. One larger area affecting most of the districts on the eastern part of the Terai and with hotspots in three of them (Sarlahi, Saptari and Sunsari), and a smaller one in the western Terai affecting other three districts, with a hotspot in Rupandehi.

*Distance to water* was the covariate with the strongest, significant, risk-increasing effect (see Table 2). For each additional kilometre from a permanent water source, the odds of snakebite increased by 1.51 times (90% CI 1.12–2.04). Similar to the human risk estimation model, here also *NDVI* had a strong effect of 0.21 (90% CI 0.05–0.86), equivalent to a snakebite odds-reducing effect of 5.76 times. Two more covariates were found to partially explain the changes in the response: *precipitation of the driest quarter* (BIO17) with a snakebite odds-reducing effect of 0.12 (90% CI 0.05–1.56) and the *annual mean temperature* (BIO1) also with an odds-reducing effect of 0.28 (90% CI 0.01–2.28). Despite not being significant, they had very important effects reducing the odds of snakebite, and their distributions, as seen in the supplementary Fig. S4, showed that their effect was unambiguous.

**Table 2:** Estimated parameters of the fitted hierarchical Bayesian model for the geospatial prediction of human snakebite risk in the Terai.

	Mean	SD	Mode	Odds ${(e}^{\mathrm{Mean}})$	Odds 90% CI	
					LL	UL
(Intercept)	0.54	1.68	0.58	1.71	0.10	26.32
NDVI	− 1.57	0.88	− 1.55	0.21	0.05	0.86
Distance water (km)	0.42	0.18	0.42	1.51	1.12	2.04
BIO1/10	− 1.29	1.04	− 1.36	0.28	0.01	2.28
BIO17/100	− 2.12	1.78	− 2.19	0.12	0.05	1.56
Range for SPDE (ρ)	28.25	10.58	23.25			
SD for SPDE (σ)	0.99	0.15	0.97			

Reporting the posterior marginal mean, standard deviation and mode (logit scale), as well as the corresponding mean, 90% lower- and upper-limit credible intervals (odds scale). The bottom rows report the spatial random effects hyperparameters. Other abbreviations: Stochastic Partial Differential Equations (SPDE).

**Figure 2:**
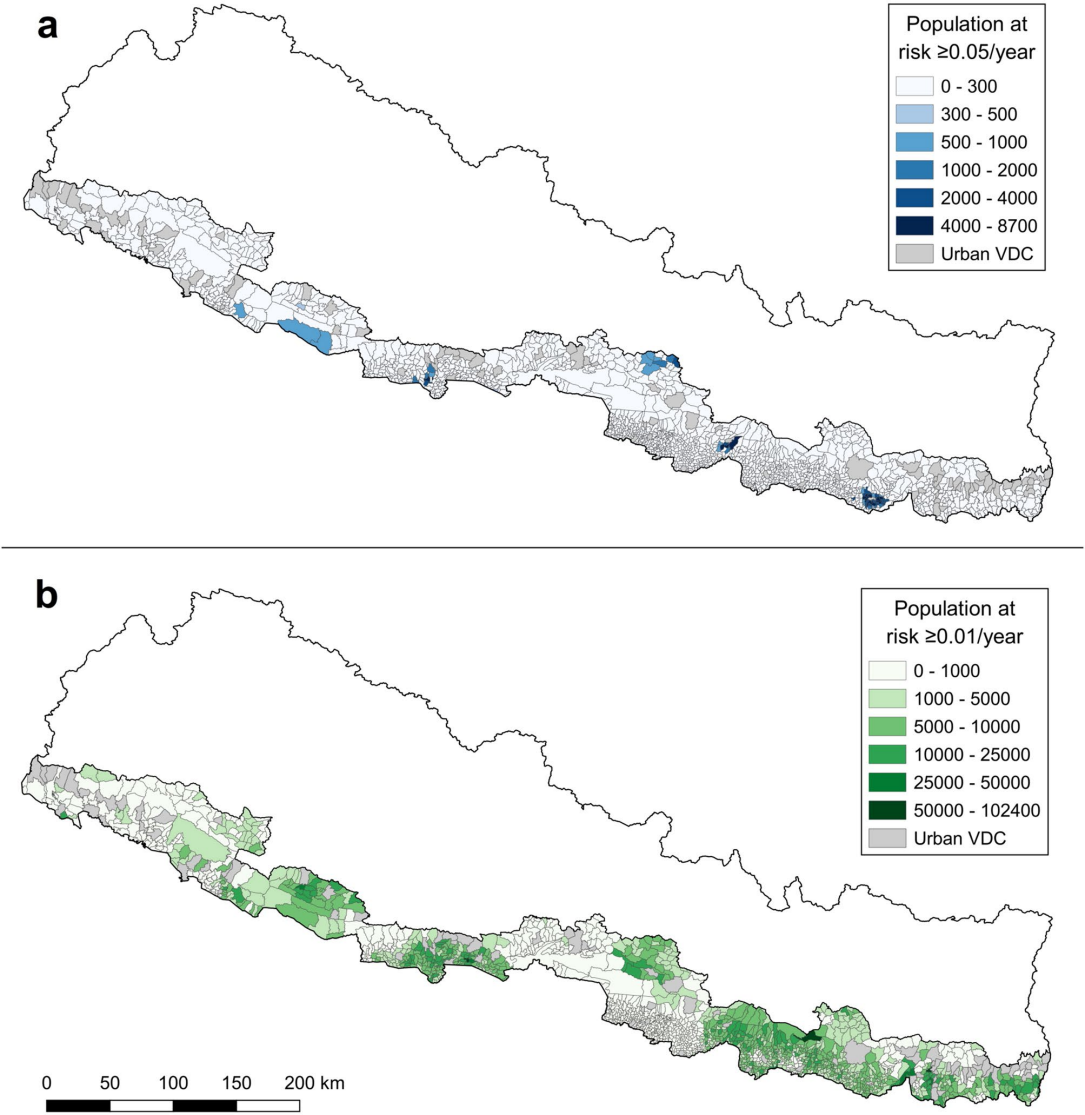
Choropleth maps aggregating the estimated population (WorldPop UN-adjusted for 2018) exposed at snakebite risks ≥ 0.05 (**a**) and (**b**) ≥ 0.01 per Village Development Committee during 12 months. (Source: vector map and administrative divisions from https://gadm.org/download_country_v3.html, projected in the local WGS 84/UTM zone 45 N coordinate reference system in QGIS 3.18.3 (https://qgis.org/en/site/)).

Based on the map of households at risk and the average number of people per household in the Terai (5.27)^9^ we extracted summary statistics for the districts’ adjusted population (i.e., not considering highly populated Village Development Committees—VDCs) living in areas at three different risk thresholds (≥ 0.01, ≥ 0.05 and ≥ 0.1, see Table 3). Choropleth maps aggregating the estimated population exposed at snakebite risks ≥ 0.01 and ≥ 0.05 per VDC during 12 months are shown in Fig. 2. Additionally, equivalent maps for municipality and district are displayed in the supplementary Figs. S5 and S6. Enlargeable, interactive versions of these maps can be found in the supplementary HTML files S1–S6. No comparable maps were plotted for the ≥ 0.1 risk, since only very few administrative units had any population over that risk threshold. We found three main predicted hotspots in the districts of Saptari, Makwanpur, and Sarlahi, where populations with at least 692 people are expected to be at an elevated snakebite risk ≥ 0.1 (see Table 3). When the risk threshold is set to a lower value (≥ 0.01), larger numbers of people in many more districts fall into that risk category, e.g., going up to 613,043 people in Rupandehi. This could represent around 6,130 possible snakebite victims in that district in 1 year. In addition to Rupandehi (91.41), six more districts in the ≥ 0.01 risk class have percentages of adjusted ‘rural’ population at risk higher than 70%, namely Saptari (86.16), Mahottari (86.11), Dhanusa (80.46), Makwanpur (73.89), Siraha (72.17), and Dang (70.79).

**Table 3:** Estimated adjusted population for 2018 in each district of the Terai (WorldPop), and population living in areas with snakebite risks larger or equal to 0.01, 0.05, or 0.1.

Region	District	Adjusted pop. (2018)	Population at ≥ 0.01 risk, (%)	Population at ≥ 0.05 risk, (%)	Population at ≥ 0.1 risk, (%)
East	Jhapa	369,014	225,609 (61.14)	0	0
East	Morang	499,966	227,215 (45.45)	24 (0.00)	0
East	Saptari	509,774	439,229 (86.16)	87,351 (17.14)	1257 (0.25)
East	Siraha	789,571	569,841 (72.17)	0	0
East	Sunsari	1,327,568	511,269 (38.51)	0	0
East	Udayapur	249,598	151,315 (60.62)	569 (0.23)	0
Central	Bara	541,445	9504 (1.76)	0	0
Central	Chitawan	329,361	7008 (2.13)	0	0
Central	Dhanusa	634,001	510,105 (80.46)	0	0
Central	Mahottari	512,858	441,618 (86.11)	109 (0.02)	0
Central	Makwanpur	256,102	189,236 (73.89)	8002 (3.12)	1025 (0.40)
Central	Parsa	1,161,894	140 (0.01)	0	0
Central	Rautahat	575,095	2136 (0.37)	0	0
Central	Sarlahi	646,882	426,967 (66.00)	24,137 (3.73)	692 (0.11)
West	Kapilbastu	581,841	256,280 (44.05)	1948 (0.33)	0
West	Nawalparasi	442,365	223,614 (50.55)	463 (0.10)	0
West	Rupandehi	670,644	613,043 (91.41)	9717 (1.45)	0
Mid-Western	Banke	330,191	125,581 (38.03)	811 (0.25)	0
Mid-Western	Bardiya	277,439	31,578 (11.38)	124 (0.04)	0
Mid-Western	Dang	396,505	280,690 (70.79)	1490 (0.38)	36 (0.01)
Mid-Western	Surkhet	157,496	52,701 (33.46)	0	0
Far-Western	Kailali	669,770	7931 (1.18)	0	0
Far-Western	Kanchanpur	728,439	22,669 (3.11)	0	0

All, the adjusted district population and the risk classes exclude the highly populated urban VDCs, removed by design, where no estimation was done.

## Discussion

Our results showed that covariates at different geographical scales (national and local) may have important effects on the risk of snakebite, both for humans and animals. The results indicate that the risk of snakebite in the Terai varies at national scale between clusters and at local scale between households. The evaluation of the final models without spatial random components and the worsening of the models’ goodness of fit as a result highlighted how snakebite risk and its determining factors are indeed spatially structured.

A strong association between high snakebite incidence and mortality, and poverty was established from the analysis of 138 countries affected by the disease^32^. In this study, we identified the *PPI*, an indicator for poverty, as a highly influential risk-increasing factor for humans. This not only confirms the critical role of poverty as a driver for this Neglected Tropical Disease, but also offers the possibility to use a standardized index at individual household scale for similar studies. Chaves et al*.*^33^ used the Poverty Gap, which is a simpler index expressing how far a person is from the average national poverty line, but to our knowledge, no study has used *PPI* for snakebite in any way. Applying *PPI* as a snakebite risk predictor also addresses previous expert calls for an Ecohealth approach to consider the relationship between the structural characteristics of houses, poverty, and snakebite^34^.

Three of the survey covariates had significant effects on the odds of snakebite. *Food storage* and *straw storage* increased them, while *sleeping on the floor* reduce them. The effect of the first two covariates is likely to be related to prey availability, represented by rodents, which are attracted by food and shelter sources. Both food and straw are very often stored near dwellings, which in the end multiply the number of possible encounters between humans, domestic animals, and the hunting snakes^20^. The expected snakebite risk reduction effect by *sleeping on the floor* is more complex though. Previously, a higher snakebite incidence was reported among rural Hindus in Maharashtra, India, due to their custom of sleeping on the floor^35,36^, while in Nepal, Chappuis et al. did not find any protective effect or significant difference in snakebite cases between sleeping on a cot or on the floor^37^. This result, nevertheless, might be influenced again by regional customs that make sleeping on the floor more common in eastern Terai (71.1% of all affirmative answers to this question), and second, by the commonly acknowledged prevalence of kraits (*Bungarus *spp.) in western Terai, which are the species most commonly linked to bites to people sleeping on the floor while hunting at night inside houses^22,38^. This geographic separation, between the human behaviour and the distribution of the species considered to cause most bites linked to it, could explain the observed shift in the odds towards a reduction effect. This effect should be further explored in localized studies designed to capture behavioural differences in humans and snakes.

For both the general human risk model and its equivalent prediction model, the covariate *Distance to water* had a significant risk-increasing effect. For each additional km in distance from permanent water sources, the odds of snakebite increased by 1.38 and 1.51 times, respectively. From a human perspective and in this socio-economic framework, it would be important to consider not only the *distance to water*, but also the path taken to get the water (or any other resource). If this path would lead a person through grasslands and open fields, this could imply an increased risk of snakebite. From an ecological perspective, there are two important aspects to consider in relation to water sources. One is, as in this study, the distance from large, constant water sources, which usually represent stable environments subject to less hydric stress. The second (not considered here) are the human-made water sources, such as ponds, reservoirs, and paddy fields that change often, are usually closer to human dwellings, and are known to attract some medically important venomous snakes (MIVS)^5^. Studies on snake migration and home range use have concluded that depending on species and ecological conditions, snakes can move between a few tens of meters per day and more than 10 km between seasons, while searching for water and prey resources^38–41^. In sub-tropical regions like the Terai, snakes living closer to continuous sources of water and vegetation should have easier access to a wider variety of prey. On the contrary, those living in agricultural areas might need to scout farther in the search for resources, encountering human-made waterbodies and prey, such as rodents^42^ and amphibians, abundant in this region^10^. Further studies considering all sources of water, and species ecology, biology and richness would be necessary to completely understand the effect of this and similar eco-physiological covariates.

Another important factor was the *NDVI*, which is a commonly used value to express photosynthetic activity, leaf production and in summary the ‘greenness’ of the environment^43^. As is the case for other covariates, its interpretation depends on the study circumstances. In Iran, it was considered an indicator of prey availability for snakes and linked to snake habitat suitability^14^. Elevated *NDVI* values have been associated with higher number of hospitalizations in Nigeria and northern Ghana, in particular during the periods of high agricultural activity, which is also related to higher snake-human contact and higher snakebite incidence^43^. In our study, its ‘protective’ effect can indeed be the consequence of better access to prey associated with healthier ecosystems, explained in the Terai by the higher *NDVI* values of the multiple dense forests distributed along the region. In addition, the averaged *NDVI* values for agricultural areas should be lower than those for perennial forests, because they include the highs and lows of production and harvest.

Environmental drivers like temperature and precipitation are common factors in geospatial analyses of snakebite^13,14,17,44^. They are found in many cases to be the main factors modulating the incidence or risk of snakebite, while varying in importance according to study conditions. For example, in Iran, *precipitation seasonality* was the most prevalent climatic covariate determining the habitat suitability leading to snakebite risk^14^, while in Mozambique, *temperature seasonality* was the predominant covariate^13^. Despite the Terai’s sub-tropical climate, the range of the average *minimum temperature of the coldest month* (BIO6) was 1.8–10.9 °C. For our snakebite risk analysis in animals, an increase of 10 °C of *BIO6* between any two points represented an increase in the odds of snakebite of 23.41 times. For snakes, this range could be the difference between total lethargy and partial activity^45^, which could lead to increased numbers of snakebites. In addition, and according to the production and holding practices of domestic animals in the Terai, this temperature range can also represent the difference between animals (mainly ruminants) being kept in sheds when at the lower range limits, or being let out of them at the upper limits, which would again increase the chances of encounters with snakes.

Similarly, for the animal model, *pig density* and *sheep density*, significantly influenced the variation in the risk of snakebite for animals in the Terai. This could be due to the conditions in which the animals and their feed are kept, favouring environments that are beneficial for either snakes or their prey. At more local scales, rather than the distribution, the presence of other animal species could instead be the factor associated with higher snakebite rates^12^. However, since the available data on domestic animal density was produced more than 10 years ago, and the animal population has grown substantially in the last years in Nepal, this outcome should be interpreted with caution.

For the animal risk, the possession of an *animal shed* also significantly increased the odds of snakebite. Similar to *straw storage*, animal sheds and similar constructions offer some shelter and at the same time attract small (prey) animals, both of which are likely to attract snakes, increasing snakebite risk for the animals using the shed. If in addition, the sheds function as poultry coops, the snake hunting behaviour might be instead targeted towards chicks and chickens^12^. Mitigation measures such as raising the coop’s floor or securing openings with fine metal mesh have been suggested to reduce this risk^12^.

The *human modification of terrestrial systems* was the only non-significant covariate in the animal risk model. However, as its strong, risk-reducing effect still seems to explain a lot of the response variation, it was retained. Its change in one unit, i.e., going from a pristine to fully modified environment, decreased the odds of snakebite by 0.13 (equivalent to 7.69 times), which agrees with previous national survey results from Sri Lanka^21^.

For our human risk prediction model, four covariates were either significant or helped to explain the changes in the response. *Distance to water* and *NDVI* were clearly significant, and *precipitation of the driest quarter* (BIO17) and *the mean annual temperature* (BIO1) helped to explain some of the response variation with convincing, unambiguous effects. For BIO17, an increase of 100 mm of rain during the driest months of the year represented an odds-reduction effect equivalent to 8.33 times. This agrees with the results of *distance to water*, suggesting that the additional availability of resources during water shortage periods, i.e., almost four times more rain (BIO17 range: 18–71 mm), could locally improve ecological conditions for snakes also leading to less scouting and fewer human encounters. Previous studies have analysed the multilevel ecological effects of droughts, e.g., reducing snake prey and leading snakes to engage in riskier behaviours^46,47^. For BIO1, the protective effect was weaker. An increase of 10 °C represented a reduction of the odds of snakebite equivalent to 3.57 times. Average temperatures for specific locations are difficult to interpret, since they might depend on mild highs and lows, strong highs and lows, or relative combinations of both. Thus, despite having a relatively important effect on the response, this effect still might be the consequence of confounding and unknown interactions.

Several other evaluated covariates, for both humans and animals, showed a negligible effect on describing the response, were not significant while having very large uncertainties, or both. Consequently, they were discarded as predicting factors. For the list of baseline covariates evaluated, see supplementary Table S1. For a complete list of available survey covariates, see Alcoba et al*.*^27^.

Some of our discarded covariates have been important in other studies, for example, to quantify snakebite risk based on reclassification methods of covariates such as *habitat suitability*, *species presence*, or *envenoming severity*^13,14,17,44,48^. These methods are especially relevant when one species (or very few) is the cause of most snakebite cases, and has differentiated optimal and sub-optimal habitats. In Nepal, and particularly in the Terai, there are at least two, and sometimes more than 10 MIVS with overlapping distributions^49^. Thus, it could be said that practically the whole region offers suitable habitat for multiple MIVS. In addition, the impossibility of reliably identifying the species having bitten the surveyed victims hindered the use of single species in the analysis. In our analysis, *species richness* was removed, as it showed almost no effect on the response. A recent meta-analysis reported an equivalent result at global scale, finding no significant difference between the number of venomous snake species in tropical and temperate locations, while the number of snakebites is clearly higher in tropical areas^50^. These results suggested that high incidence of snakebite is unrelated to *species richness*, but instead related to other factors like the number of people working in agricultural environments^21,32,50^. Another important driver of snakebite incidence has been *population density*^50^. In our study, however, any possible effect from *population density* on the risk was diminished by the random selection of households at specific numbers during study design. This was later confirmed by the minimal effect of population density as covariate in the human risk analysis.

This study presents a few limitations. For instance, despite the capacity of the INLA method to borrow strength from neighbouring observations and areas, the selection of adequate covariates with enough explanatory power still depends greatly on the number of snakebite cases, which even for a national scale study like this remains small. Also, some of the covariates with the strongest explanatory power came from our household survey, which prevented their use for generalized spatial prediction models. Concerning the animal risk analysis, due to the small number of snakebite cases we opted to aggregate all animal species and consider a grouped response. Thus, for a spatial analysis of animal risk, it was not worth it to consider each species, since that would dilute further an already sparse dataset in individual models and selection processes. Moreover, the data gathered for animals was dependent on the random selection of (human) households and unrelated to the current distribution of animal populations. This, in addition to the possible number of dry bites that go unnoticed, might be responsible for the low number of animal victims recorded (even combined across all species), making a more detailed analysis unfeasible.

Despite the large number of covariates examined during our analysis, very few were useful to predict snakebite risk along the Terai. It is possible that confounders or other difficult-to-measure covariates could better explain the complex relationship between the ecology and biology of MIVS, socio-economic factors, human behavioural traits, and the circumstances around domestic animal keeping. This needs to be further explored, following a recent call for an overarching One Health and Ecohealth approach to better understand the drivers for snakebite risk, incidence, and mortality under specific situations^34^.

In conclusion, snakebite is a multi-factorial disease and there is no possible universal approach to model its risk. Each model should be individually designed for each set of socio-economical, geographic, ecological, cultural, and environmental circumstances^19^. To better understand and address the snakebite problem, it is necessary to approach it, whenever possible, with local data collected at a national scale, so that the conclusions drawn can fuel appropriate national public health policies and actions. As long as people work, live, and keep their domestic animals in close contact with natural environments with MIVS, the risk of snakebite will be present. However, better understanding of the factors influencing that risk at the most granular scale possible, and the estimation of the populations at risk, can help to better target prevention and mitigation measures. For humans, this evidence can channel efforts towards improved access to treatment through the optimized stockpiling of antivenom, and the improvement, relocation or new construction of treating facilities, but more importantly, towards community education and sensitization in preventive campaigns^51^. Part of that preventive and educative efforts can be done at household level, by promoting and facilitating the use of protective equipment such as rubber boots, or the guidance on how to improve and adapt their immediate surroundings to make them ecologically less attractive for snakes and their prey. For domestic animals, this information could help better target awareness-raising activities for animal owners and implement mitigation strategies. For animals at higher risk, tailored interventions such as the improvement of housing conditions, regular cleaning of sheds and surrounding areas (e.g., from food waste and surrounding vegetation), and using light when animals are walked out of the enclosure at night could be deployed specifically as snakebite prevention measures^52^. It is also important to highlight that many of the factors analysed in this study affect most directly the snakes themselves, not only as snakebite agents, but also as a diverse group of species, differently affected by ecological, climatic and environmental factors in a multiplicity of settings shared with humans and domestic animals. It is therefore necessary to further investigate how those factors influence the behavioural and ecological traits of MIVS in order to truly understand this disease from a One Health viewpoint. At stake is the reduction of snakebite envenoming incidence rates in humans and animals, and of its possible long-term sequelae on human populations.

## Methods

### Area and primary data description

The geographic focus of this study was the Terai region of Nepal, populated by about 16,087,000 people (about 57.3% of Nepal’s population) for 2018 according to the UN-adjusted estimates from WorldPop^53^. In Nepal, just under 80% of the population lives in rural areas^54^ and the Terai concentrates most of these rural population^9^. Since snakebite affects primarily rural populations^32,55,56^, we started by filtering out urban and densely populated clusters areas where snakebite is considered to be unusual^21^. The primary cluster areas used for the study were the Village Development Committees (VDC), which represented the smallest, viable administrative unit recognized at the time in Nepal. Our primary data source was a multi-cluster random survey, including more than 13,800 households in all the Terai. The survey was carried out between December 2018 and May 2019, and gathered retrospectively a wide range of information in addition to the snakebite cases in humans and animals during the previous 12 months. All answers were associated to the households’ geolocation. The detailed survey methodology can be found in Alcoba et al.^27^. Two binary response variables, one for humans, and one for domestic animals, identified snakebite cases in the last 12 months (from the survey date) and defined the two main models (human and animal). Snakebite risk is defined as the theoretical probability of encountering and being bitten by a snake^51^. We considered this probability within a period of 12 months, either for a household member in the estimation model, or for anyone in a specific area of 1 km^2^ in the geospatial prediction model. In addition, snakebite risk can be measured as the likelihood of exposure to a snake (presence and abundance) times the likelihood of an encounter leading to a bite^51^. Considering the impossibility of measuring snake abundance at national scale, in this study we regressed snakebite incidence on multiple environmental and socio-economic factors to find that theoretical probability. All data management and curation, as well as the statistical analysis were done in R version 4.0.4^57^. Some input and output geospatial layers were processed in QGIS version 3.16.4^58^.

### Model architecture

The Integrated Nested Laplace Approximations (INLA) method is a computationally efficient approach for Bayesian statistical inference of latent Gaussian Markov Random Field (GMRF) models^59^. These are highly adaptable type of models including linear, generalized, mixed, spatial, and spatio-temporal models^19,60^. This type of Bayesian Hierarchical models is especially robust for the analysis of highly sparse data, such as the one presented by neglected tropical diseases like snakebite, and its integration with the Stochastic Partial Differential Equations (SPDE)^61^ allows to model multiple types of georeferenced data^19^, while borrowing strength across space and time^62^.

The models for the risk of snakebite (SB) at location $i$ satisfy the following general structure:

$${SB(s}_{i})\sim Bernoulli{(\pi (s}_{i}))$$

1 $$\begin{array}{c}logit\left({\pi (s}_{i})\right)={\eta (s}_{i})={\beta }_{0}+{{\mathbf{\rm X}(\mathbf{s}}_{i}){\varvec{\beta}}}_{j}+{u(s}_{i})\end{array}$$

$$ {u(s}_{i})\sim GMRF(\bf{0},{\varvec{\Sigma}})$$

where, β_0_ is the intercept, ${{\boldsymbol{\rm X}({\varvec{s}}}_{i}){\varvec{\beta}}}_{j}$ are the covariates’ matrix and coefficients, $i$ represents the surveyed households and their respective locations, $j$ is the number of covariates, ${\pi (s}_{i})$ is the expected value for the risk of snakebite at location $i$, and ${u(s}_{i})$ is the spatially correlated random effect. This ${\varvec{u}}$ component follows a GMRF distribution with mean zero ($\bf{0}$) and covariance matrix **Σ**, which models the data’s spatial dependency^18^. To determine ${\varvec{u}}$, it is necessary to estimate **Σ,** whose elements are defined by a Matérn covariance function (here simplified for two dimensions):

2 $$\begin{array}{c}Cov\left(U\left({s}_{i}\right), U\left({s}_{{i}^{\mathrm{^{\prime}}}}\right)\right)= {\sigma }^{2}\times \left(\kappa \times \Vert {s}_{i}-{s}_{{i}^{\mathrm{^{\prime}}}}\Vert \right)\times {\mathrm{\rm K}}_{1}\left(\kappa \times \Vert {s}_{i}-{s}_{{i}^{\mathrm{^{\prime}}}}\Vert \right)\end{array}$$

where, ${s}_{i}$ and ${s}_{{i}^{^{\prime}}}$ are spatial locations of the observations $i$ and ${i}^{^{\prime}}$, $\Vert .\Vert$ represents the Euclidean distance between two points, κ is a scale hyperparameter and K_1_ is the modified Bessel function of the second kind with order 1. For a complementary description of the INLA statistical approach, see the supplementary Document S1, and for a detailed description see Rue et al*.*^59^. The INLA method is implemented in the R package INLA (version 21.02.23)^63^.

### Selection of geospatial covariates

Two data sources were available for our analysis. The first was our national multi-cluster random survey, which provided highly granular observations for demographic, clinical, socio-economic and environmental covariates (Table 4). The second was a set of publicly available national gridded data sets (Table 4). Depending on the type of model (human or animal), we considered suitable covariates from the survey, and open-source geospatial gridded layers that could be relevant for the model response. These gridded layers were selected based on their possible relevance to snake ecology or behaviour as reported by previous studies with similar research focuses, analytical methodology, or environmental and ecological study conditions. Priority was given to studies addressing snakebite risk and the distribution of venomous snake species, often taken as a risk proxy^13–15,17,43,44^. Most of these studies based their estimations mainly on climatic covariates from the WorldClim database^64^, and on environmental covariates such as NDVI and human footprint^14^. However, since snakebite has been largely associated with poverty^32^, other covariates used to address poverty related topics, for instance illiteracy, malnutrition and filariasis^18,19,65^, were also considered.

**Table 4:** Geospatial covariates used for the current estimation of snakebite risk in humans or animals (gridded and survey based).

Category	Covariate	Description	Scaling	Data source
Environmental	NDVI annual average for 2018	Continuous	No	NEOteam
	Distance to water (Euclidean distances)	Continuous	× 0.001	Based on GeoFabrik and OSM^67^
	Human modification of terrestrial systems (HMTS) 2016	Continuous	No	NASA SEDAC^30^
Climatic	BIO6 (min. temperature of coldest month) 1970–2000	Continuous	× 0.1	WorldClim^64^
Epidemiological	Food storage	Discrete, 2 levels	No	Survey
	Straw storage	Discrete, 2 levels	No	Survey
	Sleep on floor	Discrete, 2 levels	No	Survey
Socio-economical	PPI	Continuous	× 0.01	Survey
Livestock density	Pig density, 2010	Continuous	× 0.001	Harvarddataverse^31^
	Sheep density, 2010	Continuous	× 0.001	Harvarddataverse^31^

The model selection process started with a set of 66 preliminary covariates (including available geospatial layers and relevant survey covariates), and finished with six for both the human and animal models. Highly correlated covariates were removed, first by computing the Pearson’s correlation coefficients between pairs of covariates (considering values between − 0.6 and 0.6), and second by running a sequential Variance Inflation Factor (VIF) analysis with a maximum threshold of five, also aimed at reducing multicollinearity between covariates. For each model, the selection process started with 15 to 18 covariates, which served as a baseline reference. During model selection, and after each iteration, we identified and removed parameters with posterior distribution means with negligible overall effects on the linear predictor. For that, we set a threshold between − 0.1 and 0.1 (log Odds scale), which represents an effect change of just above 10%. Additionally, we used the Watanabe-Akaike Information Criterion (WAIC) value^29^ to evaluate the goodness of fit and model iteration comparison. These two procedures helped to filter out non-significant covariates either with very small or mild effects and very large standard deviations. Similarly, we assessed the effect of interactions between several meaningful pairs of covariates in each model. Finally, we evaluated the importance of the spatial random effect, by also running each main model without that component.

### Input preparation

To avoid numerical inconsistencies due to large-scale differences between covariates, we scaled-down by factors of 10 some of the continuous covariates (see Table 4). Scaled inputs tend to work better with the default priors and to improve the performance of the models^66^. Ten of the socio-economic survey covariates were transformed into the Poverty Probability Index (*PPI*, https://www.povertyindex.org/), as intended by design. The specific questions generating this data for Nepal can be found in the supplementary Document S1. This standardized index denotes the likelihood of a household being below a certain poverty line. In this case, we used the PPI ‘100% national’ lookup table for Nepal as reference to define the poverty likelihood^28^. Finally, the covariate ‘*Distance to water’* was created by calculating Euclidean distances from each household to the nearest permanent waterbody available in OpenStreetMaps layers^67^.

### Validation

Due to the data sparseness, validation schemes such as splitting the data in training and testing subsets could not be carried out. For the risk analysis in humans, only 1.18% of the binary responses were positive, as were 0.7% in animals. Such small rates would complicate the possibility of obtaining a representative random sample, remove any power from the tests and difficult their convergence. Instead, we performed a sensitivity analysis by constantly challenging the stability of the parameters and hyperparameters under different circumstances, until no further improvement was possible. For this, we rerun tens of times each model, evaluating the effect of the addition and removal of, and interactions between, covariates via the WAIC value and the behaviour of the regression coefficients. The model runs also included the modification of the hyperparameters priors within sensible ranges for logistic regression in the SD prior (i.e., 0.1–1.5) and results from early model runs for the range prior.

### Geospatial risk prediction and differential modelling

Since the household survey covariates cannot be imputed to other locations in the Terai, for prediction purposes we implemented a variation of the human risk model. It included exclusively geospatial data available for the whole Terai region in the form of gridded data. The input data included both the survey observation points that serve again to estimate the intercept and regression parameters for the used covariates, and a regular grid of 39,684 points spaced every 1 km, covering all the Terai, where the response variable was predicted. Using the INLA methodology, a posterior distribution was approximated for each of these points, and from it the mean and standard deviation (SD) were estimated. Finally, using the mean risk and the household population density, we mapped the estimated number of households at risk of suffering a snakebite per km^2^ in 1 year. For the animal model, it was not possible to plot a snakebite risk map, since data scarcity did not allow for the spatial analysis of individual species, and a unified risk map for more than 10 species would not convey any useful information.

### Ethics statement

Ethics approval was provided by the Nepal Health Research Council (NHRC Reg. No. 585/2018), and the *Commission Cantonale d’Ethique de la Recherche Scientifique* in Geneva, Switzerland (CCER and SwissEthics Registry No. 2018-01331, Snake-Byte project). All methods were performed in accordance with the ethical guidelines from the Swiss and Nepali ethics committees for observational studies involving humans. The research project was conducted in agreement with the Declaration of Helsinki, the principles of Good Clinical Practice, the Human Research Act (HRA) and the Human Research Ordinance (HRO) as well as other locally relevant regulations. There was no experimental research carried out in animals, domestic or otherwise.

### Informed consent of human participants

The data involving human participants in this observational study was obtained through questionnaires for which written informed consent was asked to the person surveyed (or his/her legal guardian(s) in the case of minors, i.e., less than 18 years in Nepal).

## Supplementary Information

Supplementary Information 1.

Supplementary Information 2.

Supplementary Information 3.

Supplementary Information 4.

Supplementary Information 5.

Supplementary Information 6.

Supplementary Information 7.

Supplementary Information 8.

## Data Availability

The data used and the sources are described in this article and in the supplementary materials. For the primary data collected in the Snake-Byte survey, participant data that underlie the results reported in this article will be made available upon reasonable request through the University of Geneva data repository, after de-identification, beginning 12 months following the publication of this article. Requests should be directed to nicolas.ray@unige.ch.

## Code availability

The base code for reproducing the INLA analysis is available in the supplementary R-script S1.
