# Supplementary material for: Estimating and predicting snakebite risk in the Terai region of Nepal through a high-resolution geospatial and One Health approach
Source: Sci Rep. 2021 Dec 13;11:23868. doi: 10.1038/s41598-021-03301-z (PMC8668914; doi:10.1038/s41598-021-03301-z)
Supplement: Supplementary file 1 — Supplementary Information 1. [file 41598_2021_3301_MOESM1_ESM.pdf]

Supplementary information

Estimating and predicting snakebite risk in the Terai region of Nepal through a high-resolution geospatial and One Health approach

Carlos Ochoa[1,2*], Marta Pittavino[3], Sara Babo Martins[1], Gabriel Alcoba[1,4,5], Isabelle Bolon[1], Rafael Ruiz de Castañeda[1], Stéphane Joost[6], Sanjib Kumar Sharma[7], François Chappuis[5,8], Nicolas Ray[1,2]

[1] Institute of Global Health (IGH), Department of Community Health and Medicine, Faculty of Medicine, University of Geneva, Geneva, Switzerland.

[2] Institute for Environmental Sciences (ISE), University of Geneva, Geneva, Switzerland.

[3] Research Center for Statistics (RCS), Geneva School of Economics and Management (GSEM), University of Geneva, Geneva, Switzerland

[4] Médecins Sans Frontières (MSF), Geneva, Switzerland

[5] Division of Tropical and Humanitarian Medicine, Geneva University Hospitals (HUG), Geneva, Switzerland.

[6] Laboratory of Geographic Information Systems (LASIG), School of Architecture, Civil and Environmental Engineering (ENAC), École Polytechnique Fédérale de Lausanne (EPFL), Lausanne, Switzerland

[7] B.P. Koirala Institute of Health Sciences (BPKIHS), Dharan, Nepal

[8] Department of Community Health and Medicine, Faculty of Medicine, University of Geneva, Geneva, Switzerland

*Corresponding author:

Carlos Ochoa

carlos.ochoa@unige.ch

Institute of Global Health, University of Geneva

Chemin des Mines 9, 1202 Geneva, Switzerland

1. Complementary description of the INLA statistical approach

The spatial random effect $u(s_i)$ represents a normally distributed stochastic process $U(\boldsymbol{s})$, defined in a continuous domain, so it is defined as a *continuous Gaussian Random Field*. INLA introduces a Markovian process to avoid the exponential growth of the matrices involved, creating instead a sparse matrix composed mostly of zeros, and thus greatly reducing computation time. This approximates the *continuous Gaussian Random Field* by transforming it into a *discrete Gaussian Markov Random Field* and by solving, in an intermediate step, the SPDE equation:

$$W(\boldsymbol{s}) = (\kappa^2 - \Delta)^{\alpha/2} \cdot \tau \cdot U(\boldsymbol{s}) \tag{3}$$

Where, Δ is the Laplace operator, τ is the precision hyperparameter (i.e. inverse of the variance), α is a parameter controlling the smoothness of the Gaussian Random Field, and κ is the same hyperparameter defined in equation (2) in the main text.

By solving these equations, it is possible to obtain all the hyperparameters and the elements of the covariance matrix needed in the Gaussian Markov Random Field.[1] In addition, a final equation (4, the *Finite element method*) connects the spatial random effect $u(s_i)$, the SPDE output $W(\boldsymbol{s})$ and the so called *projector matrix* $\boldsymbol{A}$, which defines the observations' spatial random field by projecting them into an irregular grid or mesh (Fig. 1).

$$u(s_i) = \sum_{g=1}^{G} a_g(s_i) \times W_g = \boldsymbol{A} \times \boldsymbol{w} \tag{4}$$

Where, in addition, G is the number of vertices in the mesh, and g represents each individual vertex.

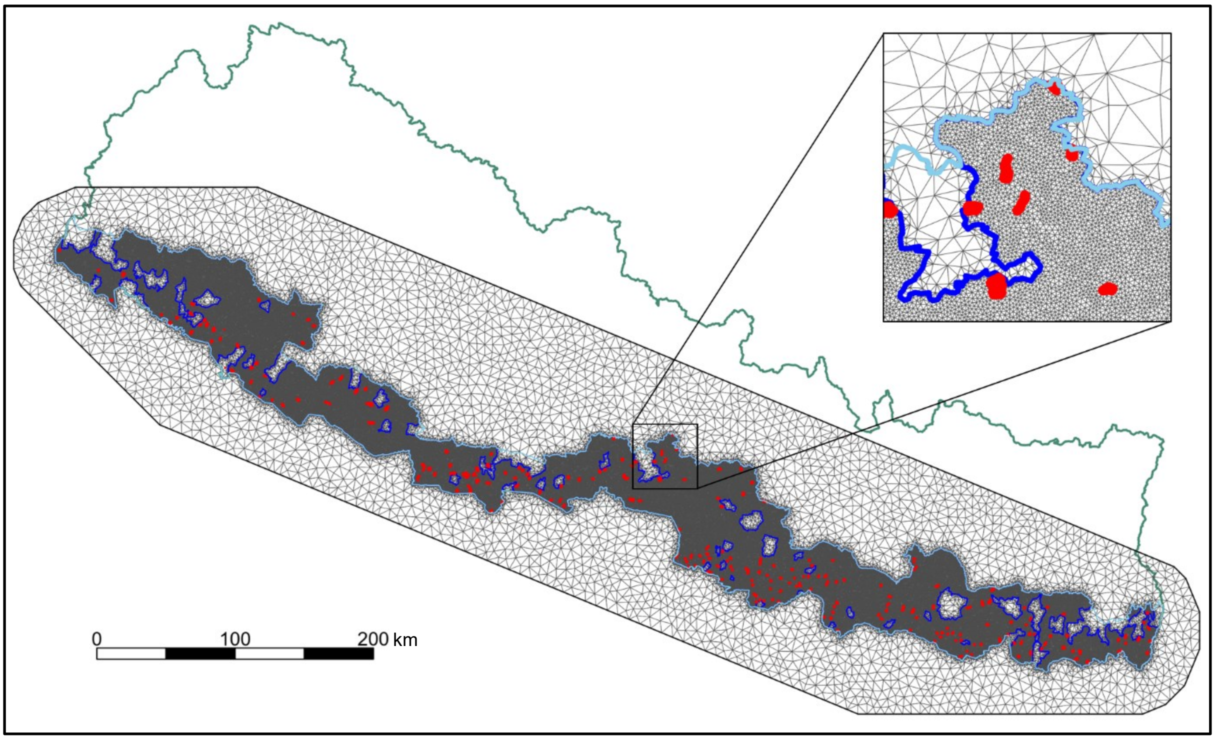

Figure 1. Mesh for the Terai (light blue line), Nepal, excluding highly populated clusters (dark blue lines). The red dots represent the observations at the selected clusters.

2. Questions generating the Probability Poverty Index for Nepal

a. How many people belong to the household?

b. In what type of job did the male head/spouse work the most hours in the past seven days?

c. How many bedrooms does your residence have?

d. What's the main construction material of outside walls?

e. What main material the roof is made of?

f. Does your residence have a kitchen?

g. What type of stove does your household mainly use for cooking?

h. What type of toilet is used by your household?

i. How many telephone sets/cordless/mobile does your household own?

j. Does your household own, sharecrop-in, or mortgage-in any agricultural land? If yes, is any of it irrigated?

3. Covariates analyzed and rejected during model selection

Table 1. Variables evaluated (after correlation and VIF analysis) and removed during model selection.

Category	Variable	Description
Climatic	WorldClim BIO3	Isothermality (BIO2/BIO7) (×100)[2]
	WorldClim BIO4	Temperature seasonality (standard deviation ×100)[2]
	WorldClim BIO11	Mean temperature of coldest quarter[2]
	WorldClim BIO14	Precipitation of driest month[2]
	WorldClim BIO15	Precipitation seasonality (coefficient of variation, average 1970-2000)[2]
	WorldClim BIO16	Precipitation of wettest quarter[2]
	Evapo-transpiration	Global Reference Evapotranspiration (Global-ET0) Version 2[3]
	Aridity	Global Aridity Index (Global-Aridity_ET0)[3]
Socio-economic	Risk of outer toilet	Survey question on household risk factors: toilet outside the household
	Risk of open defecation	Ditto: open field defecation or urination
	Risk by not use of bed nets	Ditto: use of mosquito nets to sleep at night
	WorldPop PPP 2018	People per pixel (population density) for Nepal in 2018[4]
Ecological	Species richness	Number of species of medically important venomous snakes[5]
	Household environment	Survey question on the type of environment surrounding the household
Geographic	Altitude	Digital Elevation Model (DEM)[6]
	Slope	Terrain inclination derived of the DEM in percentage
Domestic animals	Number of cattle/buffalos	Survey question on livestock and domestic animals possessed during the last year
	Number of horse/donkey	Ditto
	Number of goats	Ditto
	Number of sheep	Ditto
	Number of pigs	Ditto
	Number of poultry	Ditto
	Number of dogs	Ditto
	Number of cats	Ditto
	Number of other animals	Ditto
	Cont. poultry density	Global distribution data for chickens and ducks in 2010[7]
	Cont. goats density	Global distribution data for goats in 2010[7]

4. Marginal posterior distributions of parameters and hyperparameters

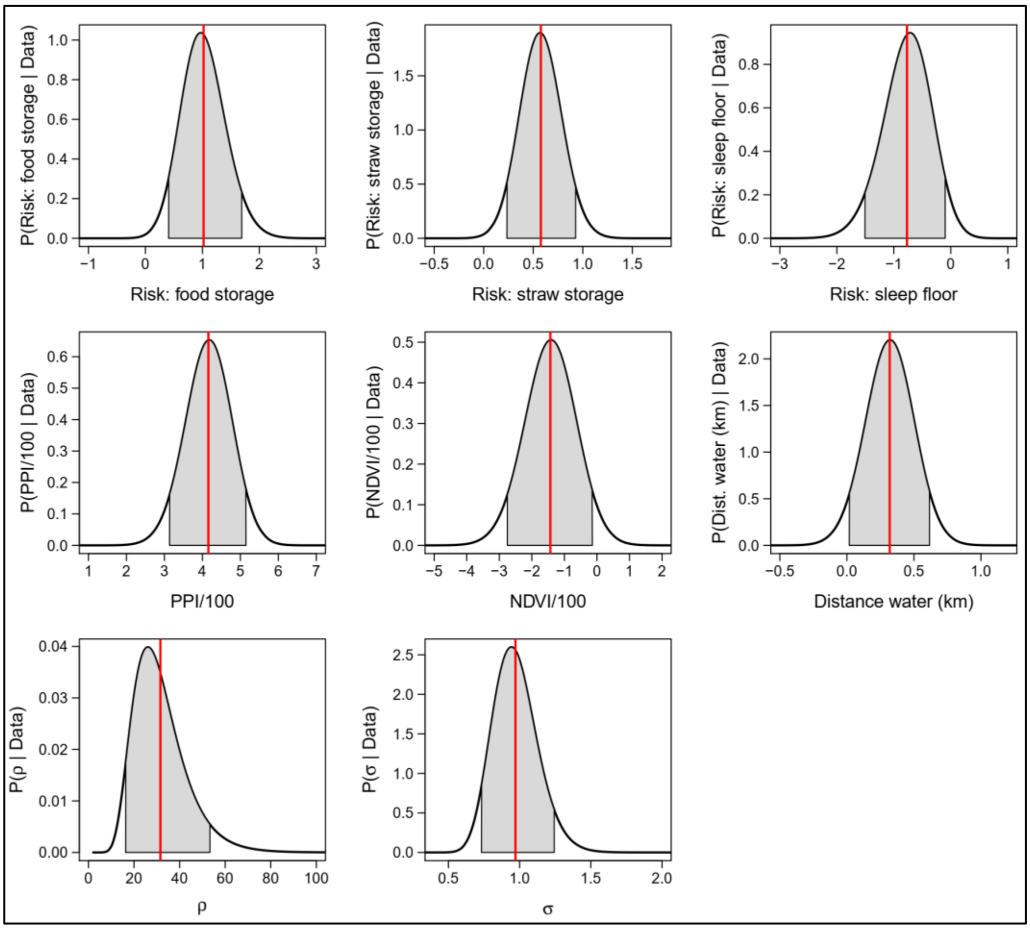

Figure 2. Posterior distribution of the coefficients for the human risk estimation parameters (and hyperparameters (ρ and σ). The grey area represents the 90% CI and the red line indicates the mean.

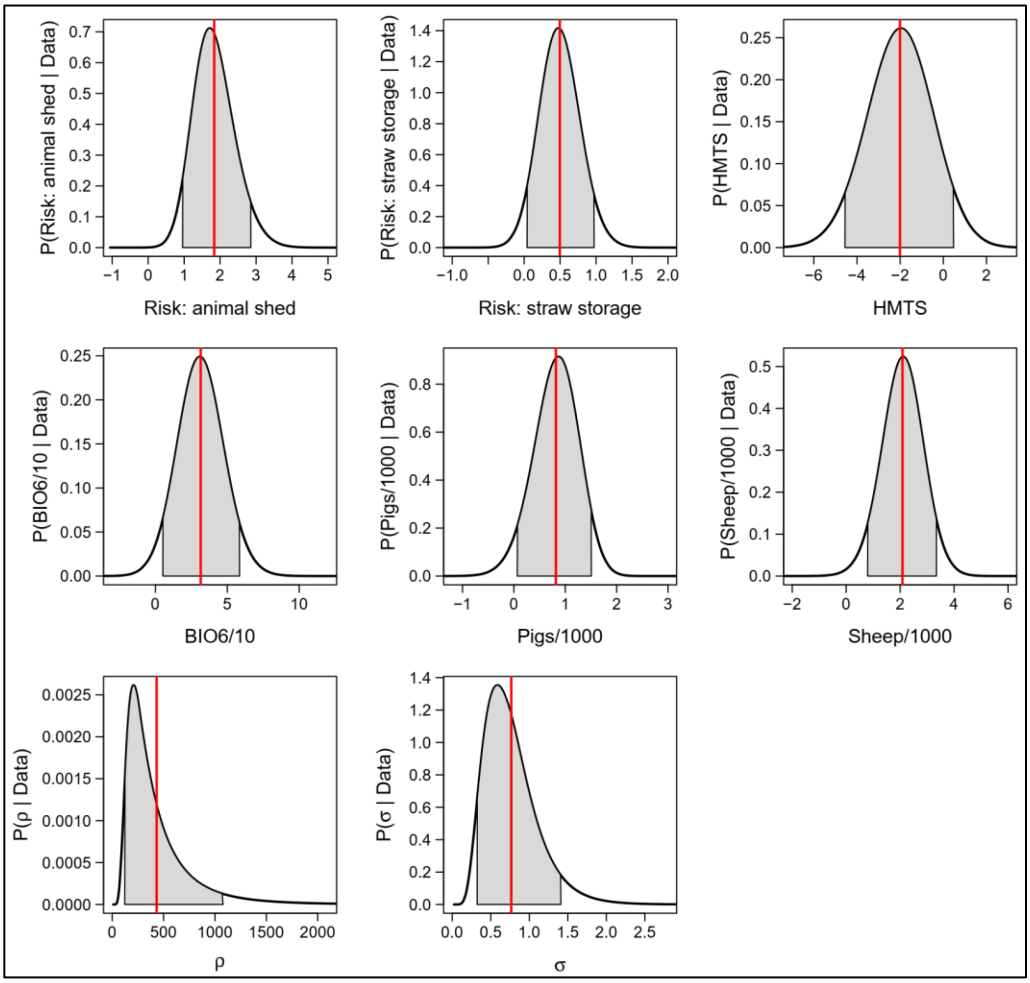

Figure 3. Posterior distribution of the coefficients for the animal-risk estimation parameters and hyperparameters (ρ and σ). The grey area represents the 90% CI and the red line indicates the mean.

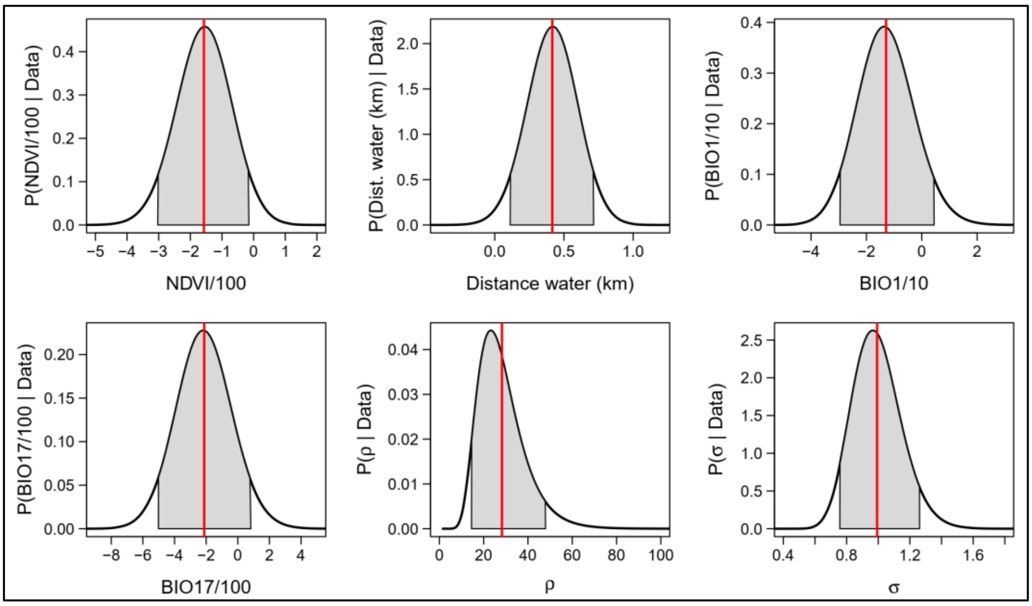

Figure 4. Posterior distribution of the coefficients for the human risk estimation and prediction parameters and hyperparameters (ρ and σ). The grey area represents the 90% CI and the red line indicates the mean.

5. Choropleths maps of population at risk

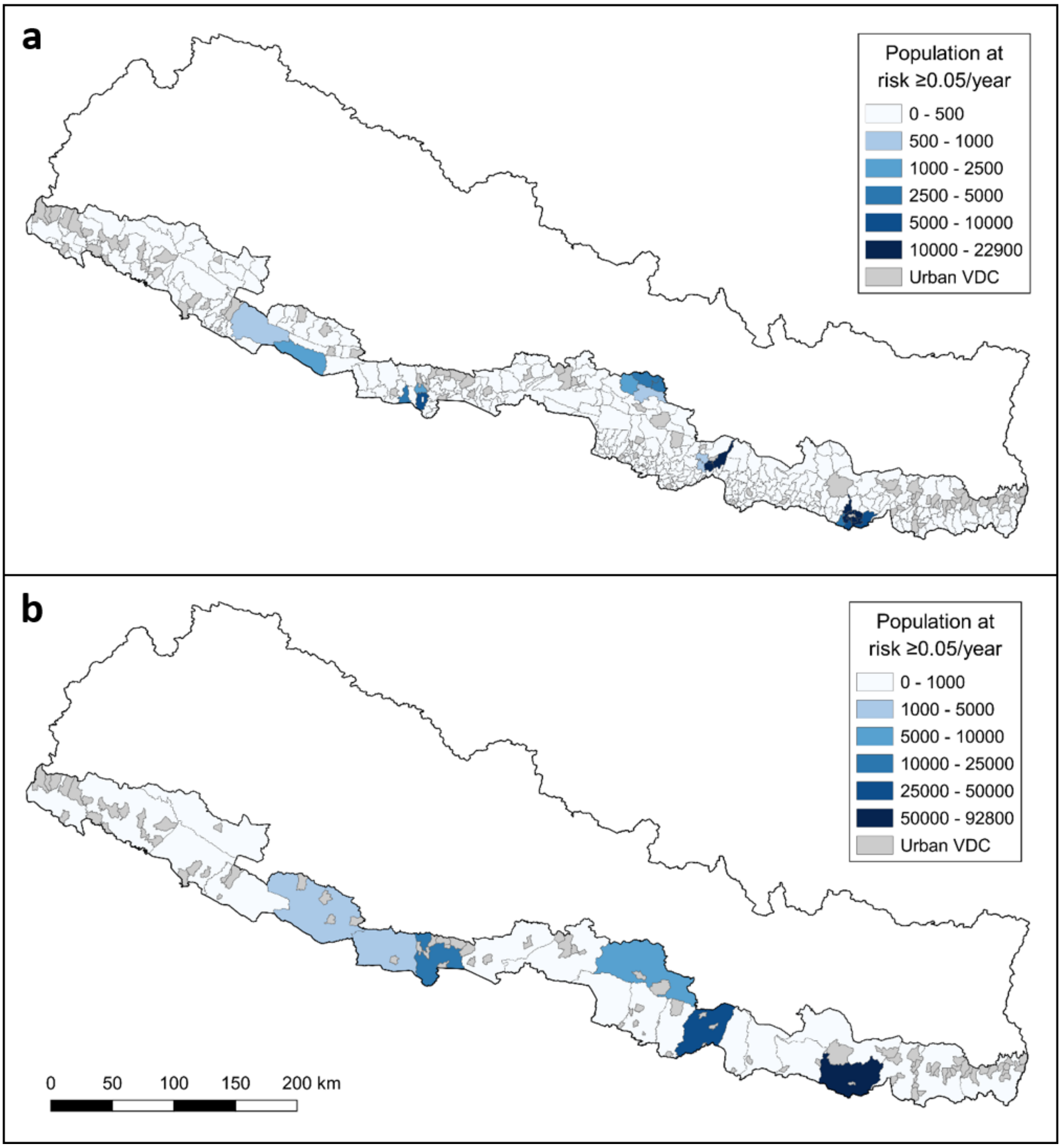

Figure 5. Estimated population living in areas with a risk of snakebite larger than 0.05 per municipality (**a**) and district (**b**) during 12 months. The grey areas represent the highly populated VDCs removed by design, where no estimation was done.

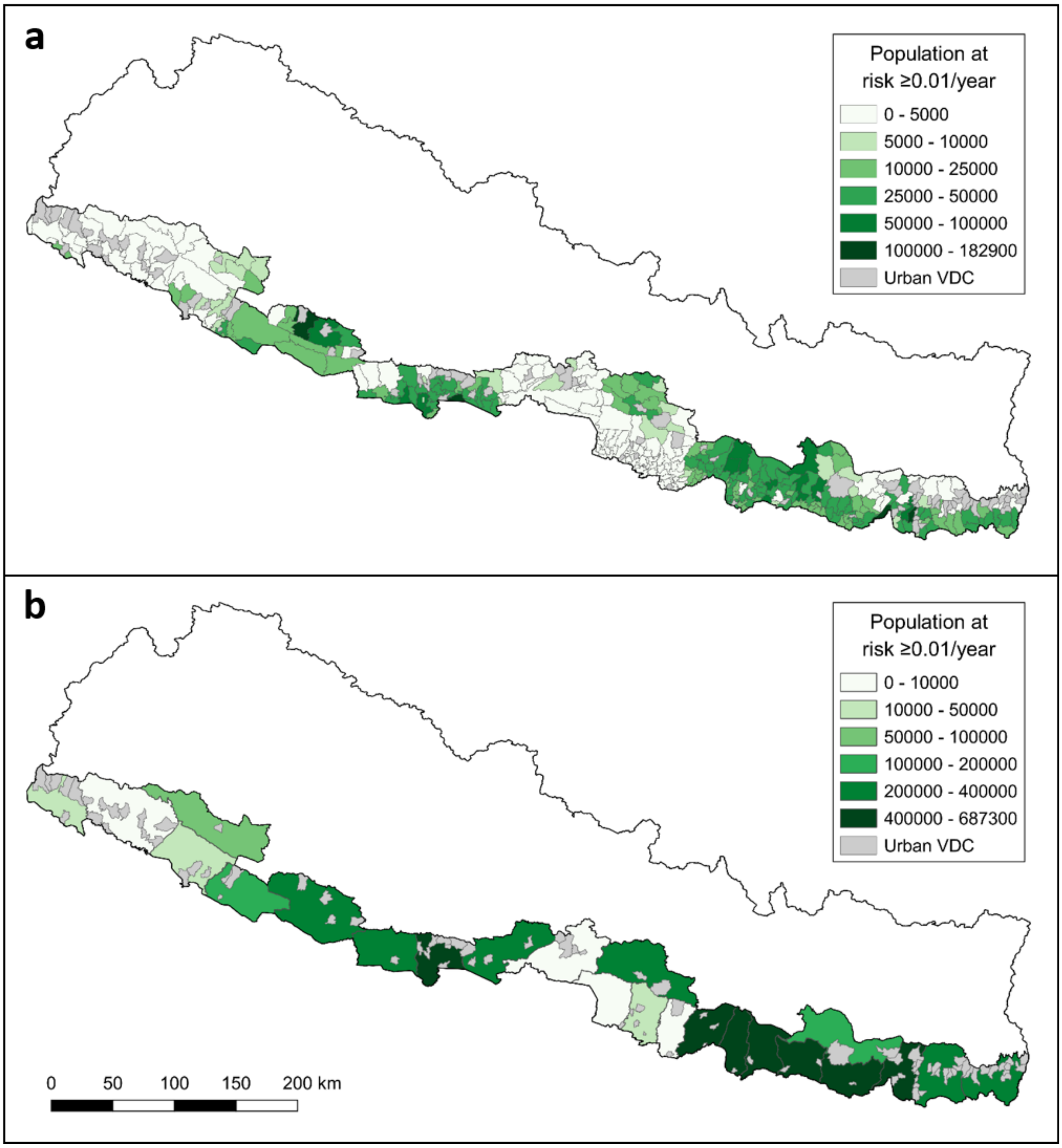

Figure 6. Estimated population living in areas with a risk of snakebite larger than 0.01 per municipality (**a**) and district (**b**) during 12 months. The grey areas represent the highly populated VDCs removed by design, where no estimation was done.

6. References to the supplementary information

1 Lindgren, F., Rue, H. & Lindström, J. An explicit link between Gaussian fields and Gaussian Markov random fields: the stochastic partial differential equation approach. *Journal of the Royal Statistical Society: Series B (Statistical Methodology)* **73**, 423-498 (2011).

2 Fick, S. E. & Hijmans, R. J. WorldClim 2: new 1-km spatial resolution climate surfaces for global land areas. *International journal of climatology* **37**, 4302-4315 (2017).

3 Trabucco, A. & Zomer, R. J. Global aridity index and potential evapotranspiration (ET0) climate database v2. *CGIAR Consort Spat Inf* (2018).

4 WorldPop. Global high resolution population denominators project funded by the Bill and Melinda Gates Foundation (OPP1134076). *School of Geography and Environmental Science, University of Southampton; Department of Geography and Geosciences, University of Louisville; Departement de Geographie, Universite de Namur, and Center for International Earth Science Information Network (CIESIN), Columbia University (2018)*, doi:https://dx.doi.org/10.5258/SOTON/WP00675 (2020).

5 Roll, U. *et al.* The global distribution of tetrapods reveals a need for targeted reptile conservation. *Nature Ecology & Evolution* **1**, 1677-1682 (2017).

6 Farr, T. G. *et al.* The shuttle radar topography mission. *Reviews of geophysics* **45** (2007).

7 Gilbert, M. *et al.* Global distribution data for cattle, buffaloes, horses, sheep, goats, pigs, chickens and ducks in 2010. *Scientific data* **5**, 1-11 (2018).
